# Echocardiographic validation of pulmonary hypertension due to heart failure with reduced ejection fraction in mice

**DOI:** 10.1038/s41598-018-19625-2

**Published:** 2018-01-22

**Authors:** Nour R. Dayeh, Jean-Claude Tardif, Yanfen Shi, Mégane Tanguay, Jonathan Ledoux, Jocelyn Dupuis

**Affiliations:** 1 Research Center, Montreal Heart Institute, Quebec, Canada; 2 Department of Medicine, Université de Montréal, Quebec, Canada; 3 Department of Pharmacology and Physiology, Université de Montréal, Quebec, Canada

## Abstract

Pulmonary hypertension (PH) associated with left heart diseases is the most prevalent cause of PH. The scarcity of studies exploring the pathophysiology and therapies of group II PH resides in the lack of validated small animal models with non-invasive determination of the presence and severity of PH. Heart failure (HF) was induced in mice by coronary artery ligation. Mice developed PH as evidenced by an elevated right ventricular (RV) systolic pressure and RV hypertrophy. Detailed non-invasive echocardiographic analysis on the left and right ventricles showed impaired left ventricular (LV) systolic and diastolic function. In addition, RV hypertrophy was confirmed by echo and accompanied by impaired function as well as increased pulmonary resistance. Correlation analysis validated the use of the LV wall-motion score index (WMSI) at a threshold value of ≥2.0 as a powerful and reliable indicator for the presence of PH and RV dysfunction. Echocardiography is an accurate non-invasive technique to diagnose PH in a HF mouse model. Moreover, an echocardiographic parameter of infarct size and LV function, the LV WMSI, reliably correlates with the presence of PH, RV hypertrophy and RV dysfunction and could be used to improve efficiency and design of pre-clinical studies.

## Introduction

The prognosis for left heart diseases in patients with preserved or reduced ejection fractions with associated pulmonary hypertension (PH) is very poor^1^. Additionally, PH associated with left heart diseases (LHD), classified as Group II PH, is the most prevalent form of PH^1^, thus confirming the need for adequate therapeutic options. Group II PH is defined as a mean pulmonary arterial pressure ≥25 mmHg at rest associated with a pulmonary capillary wedge pressure >15 mmHg^2^. While potential therapeutic options have been suggested by clinical studies testing treatments for group I PH (PAH)^3–8^, there is currently no approved treatment for group II PH. However, the pathophysiology of Group II PH is distinct from group I PH and few studies focused on group II PH using animal models^9–22^. The limited pre-clinical literature on Group II PH is in part due to the technical burden of the assessment of hemodynamic alterations.

Myocardial infarction is the most frequent cause of heart failure with reduced ejection fraction (HFrEF). The coronary artery ligation model is commonly used in animals to develop HFrEF with usual emphasis on the study of LV function. Few studies have however used that model to study group II PH. The development of PH in this model is essentially dependant on the severity of LV dysfunction that in turn will vary with infarct size, resulting in no or variable severity of PH. There has been no validation of proper diagnostic tools that can be utilized to non-invasively determine or predict the presence and severity of PH in those animals. Investigators usually use invasive hemodynamic evaluation with right heart catheterization to determine the presence of PH^9–11^. However, considering chamber size, right ventricular catheterization is a technically challenging invasive and usually terminal method. Alternatively, echocardiography is an attractive non-invasive method in the detection of pulmonary arterial hypertension (PAH) and right ventricular hypertrophy^23^. In humans, echocardiographic variables clinically used for the diagnosis of left heart failure, pulmonary hypertension and right heart function are well defined^24^. Surprisingly, corresponding characterization has yet to be performed in the mouse model of HFrEF induced by myocardial infarction despite the substantial advantages of echocardiography such as being non-invasive, allowing potential repetitive measurements to monitor progression of PH and identification of predictive PH severity in early stages of the disease.

The purpose of this study is to validate, through the use of echocardiography, the murine myocardial infarction HFrEF model for the study of WHO group II PH. In addition, we aimed to determine the possibility to use echocardiographic measurement of infarct size by the LV wall motion score index (WMSI) as a predictive parameter of the development of PH in this pre-clinical model.

## Methods

The study was performed according to procedures approved by the ethics committee for animal research of the Montreal Heart Institute in accordance with the Canadian guidelines for the care of laboratory animals.

### Surgical procedures

A total of 67 mice aged 2 to 3 months (male and female, C57BL6 background, body weight 19–25 g) were used. Under anaesthesia, animals of the PH group (n = 48) underwent thoracotomy followed by ligation of the left anterior descending coronary artery causing myocardial infarction (MI) as carried out previously in rats^9,15^. A post-MI survival rate of 50% due to early mortality within the first week was noted (Supplemental Fig. 1) and is consistent with other rodent models^25,26^. Animals of the sham group (n = 19) underwent lateral thoracotomy without ligation of the coronary artery without any premature death.

### Hemodynamic measurements and respiratory function tests

Pulmonary and hemodynamic parameters were evaluated 4 weeks after surgery. Mice were anesthetized with xylazine (10 mg/kg) and ketamine (50 mg/kg). The trachea was isolated and connected to a ventilator (Flexivent, SCIREQ, Canada) to assess respiratory function. Measurement of hemodynamic parameters was performed using high-fidelity catheters (1.4 F) to measure intraventricular pressure (Millar Instruments, USA). Briefly, the catheter is inserted in the right carotid artery and advanced into the left ventricle (LV), or inserted in the right jugular vein and advanced into the right ventricle (RV) to monitor LV and RV hemodynamic parameters, respectively. Measurements were made on a polygraph (Powerlab, AD Instruments, USA).

### Morphometric measurements of the heart and lungs

Following pulmonary and hemodynamic evaluation, lungs and hearts were harvested and dissected. Right ventricular hypertrophy was assessed by the Fulton index (RV/(LV + septum) weight). Scars were dissected from the LV and weighed. Scar surface measurements were conducted by planimetry. To determine pulmonary structural remodelling, the wet lung (WL) weight and dry lung (DL) weight were measured and ratios WL/body weight (BW), and DL/BW were calculated. The left lung was perfused and fixed with optimal cutting temperature compound (OCT) and one lobe was removed and placed in cassettes left in formalin. Paraffin embedded histological slides from lung tissue of MI and Sham mice were stained with Masson’s trichrome and collagen deposition in the lungs was evaluated by histologic quantification.

### Echocardiographic measurements

All animals had complete echocardiographic data. Transthoracic echocardiography was performed prior to terminal assessment of cardiac and pulmonary functions using an i13L probe (10–14 MHz) and Vivid 7 Dimension ultrasound system (GE Healthcare, Norway), with mice being lightly sedated by 1.5–2% isofluorane. An average of 3 consecutive cardiac cycles was used for each measurement.

Two-dimensional echocardiography was used to visualize left ventricular (LV) wall segment’s motion. 10 segments in short axis views (6 at level of papillary muscle and 4 at apex) were visualized for wall motion scoring. They were readably recorded in all mice scanned. For each myocardial segment, LV wall motion was scored as normal (1), hypokinesis (2), akinesis (3), dyskinesis (4), or aneurysmal (5). The LV Wall motion score index (WMSI) was calculated as sum of all scores/number of segments analysed. Thickness of LV anterior and posterior walls at end cardiac diastole (LVAWd, LVPWd), LV dimensions at end cardiac diastole and systole (LVDd, LVDs) were measured by M-mode echocardiography (M-mode). LV fractional shortening (FS), LV volumes at end cardiac diastole and systole (LVVd, LVVs) were determined and the LV ejection fraction computed from the Vivid 7 system software: FS = (LVDd − LVDs)/LVDd × 100%, EF = (LVVd − LVVs)/LVVd × 100%. LV mass was calculated using a formula recommended by Liao Y *et al*.^27^. Time interval from mitral valve closure to opening (MVCO) was measured in trans-mitral flow by pulsed wave Doppler (PW). LV ejection time (LVET) was measured from the beginning to the ending of flow in LV outflow tract (LVOT) obtained also by PW. LVOT flow was traced to obtain cardiac output (CO). LV global myocardial performance index (MPI global) was calculated as (MVCO−LVET)/LVET × 100%.

Right ventricular (RV) anterior wall thickness (RVAWd) and RV dimensions at end-diastole (RVDd) were measured by M-mode echocardiogram in parasternal long axis view at the level of aortic valve. Tricuspid annulus plane systolic excursion (TAPSE) was measured by M-mode. Pulmonary artery acceleration time (PAAT), and ejection time (PAET) were measured from pulmonary artery flow obtained by PW. Tricuspid annulus moving velocity in systole (S_R_) was measured by tissue Doppler imaging.

The datasets generated during and/or analyzed during the current study are available from the corresponding author on reasonable request.

### Statistical analysis

Statistical analsis was performed using GraphPad Prism 7 software. Experimental groups were analyzed by ANOVA followed, when a significant group interaction was found, by multiple comparisons with Tukey’s posthoc analysis. Values are presented as mean ± SE. WMSI correlations with hemodynamic and echocardiographic variables was obtained by linear regression analysis. Significant values were considered at p < 0.05.

## Results

### WMSI as a marker of LV dysfunction

**Figure 1:**
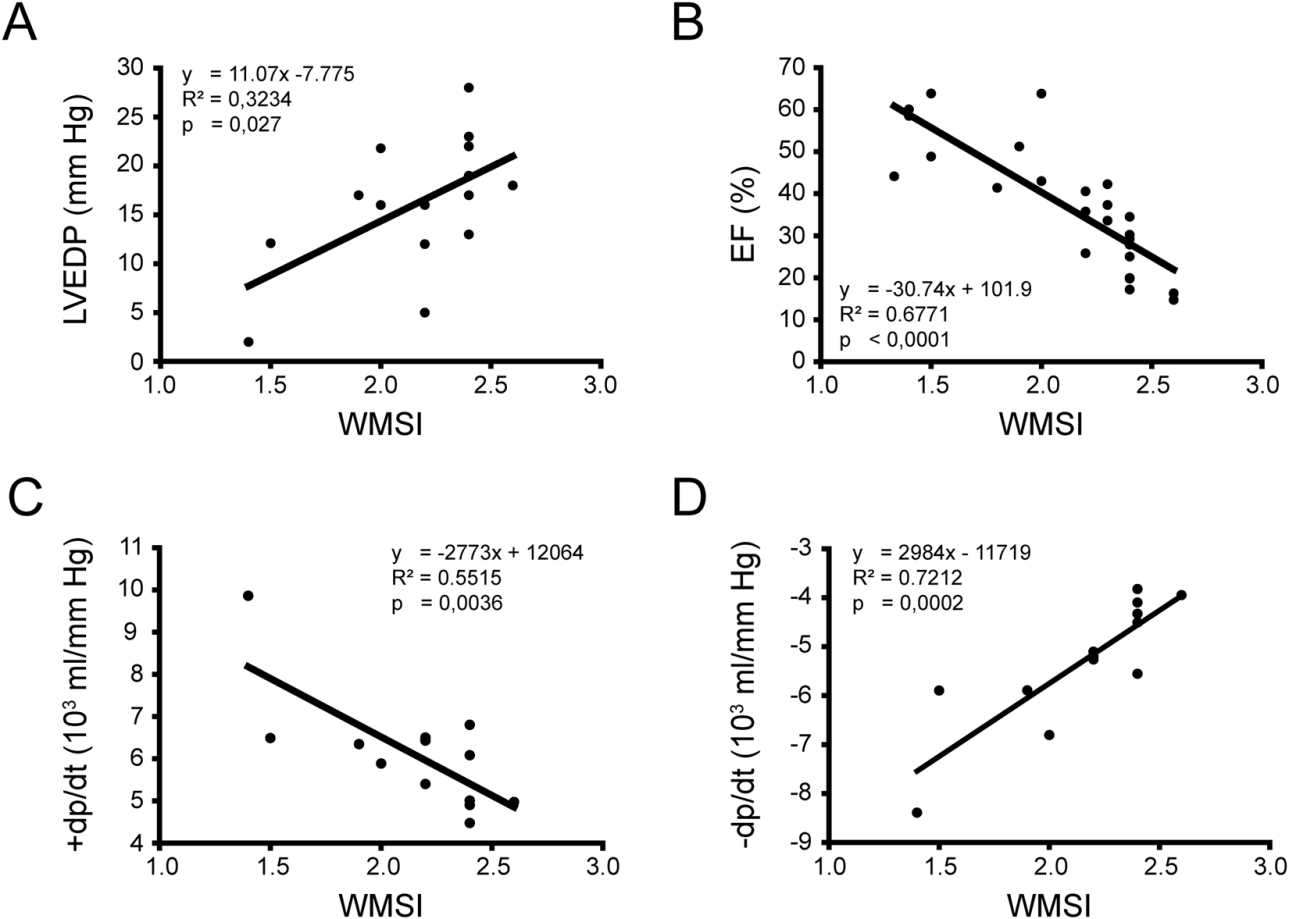
Left ventricular function and Wall Motion Score Index. Correlation between Wall Motion Score Index (WMSI) and different left ventricular function parameters in mice following coronary ligation. Left ventricular function was assessed with left ventricular end diastolic pressure (LVEDP; panel A; n = 15), left ventricular ejection fraction (EF; panel B; n = 25), left ventricular inotropy (+dP/dt; panel C; n = 13) and left ventricular lusitropy (−dP/dt; panel D; n = 13).

From a hemodynamic standpoint, ischemia-induced LV dysfunction is characterized by an increased LV end-diastolic pressure (LVEDP) as well as impaired rates of ventricular contraction (+dP/dt) and relaxation (−dP/dt), leading to a reduced ejection fraction. Alteration of mouse LV function following coronary ligation was thus assessed by hemodynamic monitoring through intraventricular catheterization and echocardiography measurements. The hemodynamic parameters were analyzed with respect to WMSI values determined by echocardiography. LVEDP (R^2^ = 0.3234; p = 0.0270) and LV ejection fraction (R^2^ = 0.6771; p < 0.0001) correlated with WMSI (Fig. 1A,B). Accordingly, WMSI values were found to correlate strongly with LV contractile (R^2^ = 0.5515; p = 0.0036) and relaxation rates (R^2^ = 0.7212; p = 0.0002) (Fig. 1C and D).

### WMSI and LV infarct sizes

Highest probability of developing PH was obtained in this investigation by focusing on mice with medium to large sized infarcts. Upon coronary ligation, myocardial scar surfaces ranged from 0 mm^2^ (no scar) to 55 mm^2^ as measured by pathologic planimetry. Furthermore, scar dimension and the associated dysfunctional LV were also sought non-invasively by correlation with WMSI.

**Figure 2:**
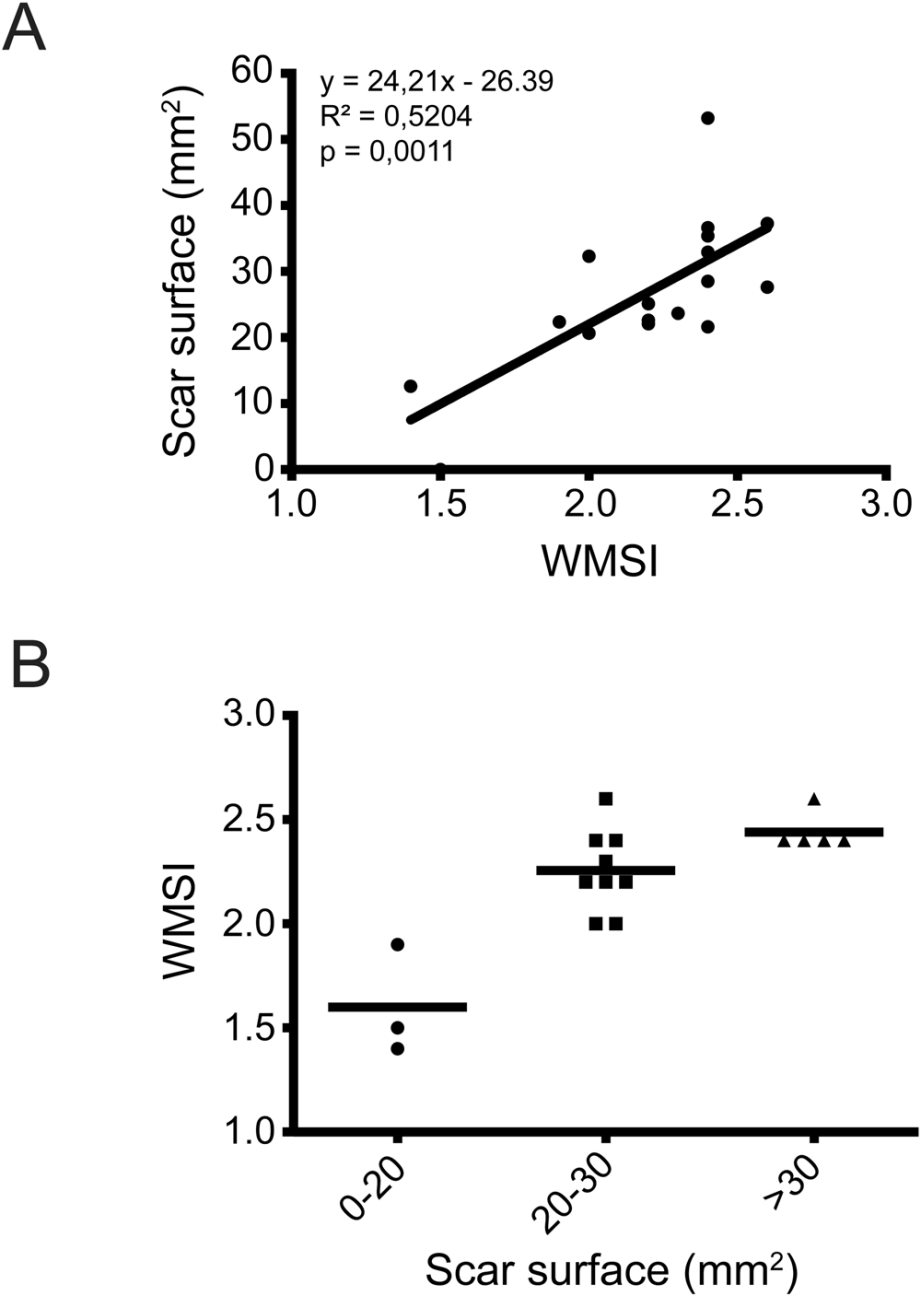
Wall Motion Score Index and infarct size. (**A**) Correlation between Wall Motion Score Index (WMSI) and the extent of infarct (scar surface) induced by coronary ligation in mice. (**B**) WMSI correlation with scar surface illustrates that a WMSI ≥ 2.0 corresponds to a scar surface of ≥20 mm^2^. n = 17.

Mice were sorted based on scar surface area and compared to WMSI values. WMSI and scar surface values correlated positively (R^2^ = 0.5204; p = 0.0011) (Fig. 2A). All mice with medium to large infarcts had WMSI values superior or equal to 2 (Fig. 2B). Consequently, a WMSI threshold value of 2 was set for post-surgery identification of mice with significant infarct surfaces and LV dysfunction and therefore at greater likelihood of developing PH and RV dysfunction.

### Echocardiographic assessment of LV dysfunction and RV hypertrophy in mice with WMSI ≥2.0

**Figure 3:**
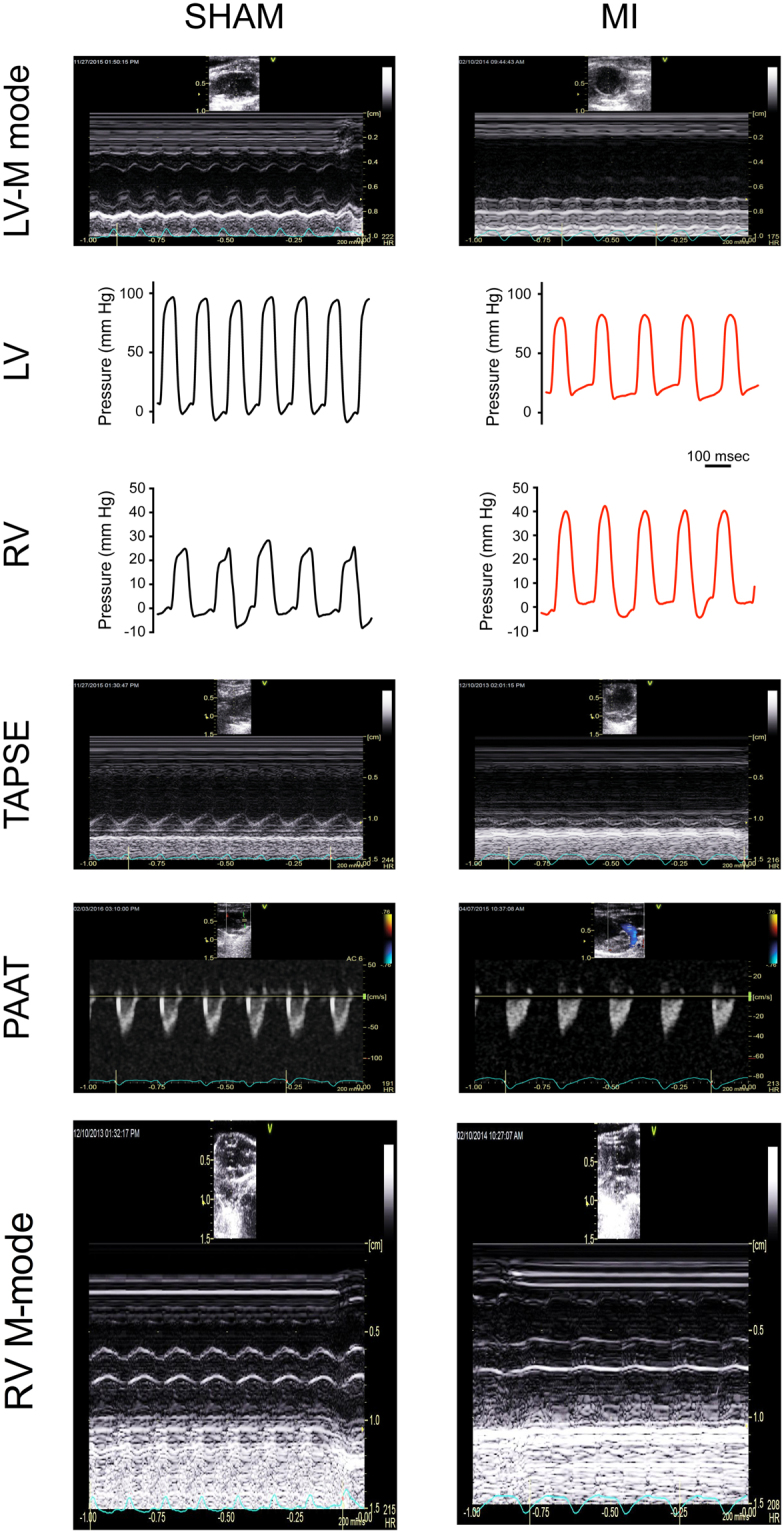
Echocardiographic and hemodynamic monitoring of cardiac function. Typical examples of cardiac function monitoring in mice without (SHAM, left column) or following coronary ligation (MI, right column). LV-M mode: M-mode echocardiographic recordings of left ventricular cycle. LV, RV: Intraventricular pressure measured in left (LV) and right (RV) ventricles with high-fidelity catheters. TAPSE: Echocardiographic assessment of tricuspid annular place systolic excursion (TAPSE). PAAT: Echocardiographic evaluation of pulmonary artery acceleration time (PAAT).

In mice with WMSI values ≥2.0, in depth echocardiographic analysis revealed LV dysfunction (Fig. 3). An increase in LV size was noted concomitantly with impaired LV systolic function and LV myocardial performance (MPI_Global_). In addition, echocardiography revealed RV hypertrophy (end-diastolic anterior wall thickness (RVAW_d_)) and dilation (end-diastolic dimension (RVD_d_)) coinciding with impaired RV systolic function (tricuspid annulus plane systolic excursion (TAPSE)). Pulmonary hypertension was also evidenced by indicators of increased RV afterload with lower values of pulmonary artery acceleration time (PAAT) and pulmonary artery ejection time (PAET) (Table 1). Non-simultaneous measurements of PAAT at echo and of RVSP by hemodynamics were available for 15 animals and did not show correlation (R^2^ = 0.11, p = 0.23), emphasizing the technical limitations of hemodynamic measurements in small animals. Details of the wall motion abnormalities form all of the myocardial segments analyzed (10 per animal) is presented in Supplemental Table 1.

**Table 1:** Echocardiographic evaluation of ventricles in SHAM and MI mice.

**Parameters**	**Sham n = 19**	**MI (WMSI < 2) n = 7**	**p value WMSI < 2 vs sham**	**MI (WMSI ≥ 2) n = 18**	**p value (WMSI ≥ 2 vs sham)**	**p value WMSI < 2 vs WMSI ≥ 2**
**Left ventricular dimensions**						
Anterior wall thickness at end diastole (LVAWd, mm)	0.72 ± 0.01	0.56 ± 0.05	n.s.	0.44 ± 0.06	<0.0001	n.s.
Posterior wall thickness at end diastole (LVPWd, mm)	0.7 ± 0.01	0.75 ± 0.01	n.s.	0.8 ± 0.03	<0.001	n.s.
Dimension at end diastole (LVDd, mm)	3.8 ± 0.06	4.5 ± 0.18	<0.05	5.2 ± 0.16	<0.0001	<0.05
Dimension at end systole (LVDs, mm)	2.35 ± 0.07	3.47 ± 0.18	<0.01	4.57 ± 0.2	<0.0001	<0.01
Volume at end diastole (LVVd, ml)	0.14 ± 0.006	0.23 ± 0.03	n.s.	0.36 ± 0.03	<0.0001	<0.05
Volume at end systole (LVVs, ml)	0.04 ± 0.003	0.11 ± 0.02	n.s.	0.26 ± 0.03	<0.0001	<0.01
**Left ventricular function**						
Wall motion score index (WMSI)	*1*	*1,55* ± *0.08*	<0.0001	*2.33* ± *0.08*	<0.0001	<0.0001
Ejection fraction (%)	74 ± 2	53 ± 3	<0.0001	31 ± 3	<0.0001	<0.0001
Cardiac output (ml/min)	12.4 ± 0.4	11.3 ± 0.7	n.s.	8.7 ± 0.6	<0.0001	<0.05
Fractional shortening (%)	38 ± 1	23 ± 2	<0.0001	13 ± 1	<0.0001	<0.01
Myocardial performance index (MPI, %)	47.52 ± 2.6	59.55 ± 7.8	n.s.	93.76 ± 5.3	<0.0001	<0.001
**Left Atrial dimensions and function**						
Left Atrial dimensions (mm)	2.32 ± 0.24	2.41 ± 0.25	n.s.	3.02 ± 0.4	<0.0001	<0.001
E/e′	35.2 ± 6.79	36.9 ± 5.1	n.s.	75.6 ± 1.65	<0.0001	<0.0001
**Right ventricular dimensions and function**						
Anterior wall thickness at end diastole (RVAWd, mm)	0.31 ± 0.01	0.38 ± 0.03	<0.05	0.38 ± 0.02	<0.01	n.s.
Dimension at end diastole (RVDd, mm)	1.74 ± 0.05	1.85 ± 0.06	n.s.	2.15 ± 0.1	<0.01	n.s.
Tricuspid annulus plane systolic excursion (TAPSE, mm)	1.06 ± 0.04	0.95 ± 0.04	n.s.	0.76 ± 0.04	<0.0001	n.s.
Lateral wall systolic contractility (S_R_, cm/sec)	3.25 ± 0.25	3.04 ± 0.23	n.s.	2.1 ± 0.12	<0.001	<0.05
**Pulmonary Artery**						
Pulmonary artery acceleration time (PAAT, msec)	20.18 ± 0.76	16.75 ± 1.1	<0.05	15.62 ± 0.49	<0.0001	n.s.
Pulmonary artery ejection time (PAET, msec)	67.52 ± 2.09	63.17 ± 3.3	n.s.	60.31 ± 1.6	<0.05	n.s.
PAAT/PAET	0.3008 ± 0.01	0.27 ± 0.02	n.s.	0.26 ± 0.01	n.s.	n.s.

LVAWd: left ventriclular anterior wall thickness in diastole, LVPWd: left ventriclular posterior wall thickness in diastole, LVDd: left ventriclular dimension in diastole, LVDs: left ventriclular dimension in systole, LVVd: left ventriclular volume in diastole, LVVs: left ventriclular volume in systole, WMSI: wall motion score index, EF: ejection fraction, CO: cardiac output, FS: fractional shortening, MPI: myocardial performance index, RVAWd: right ventriclular anterior wall thickness in diastole, RVDd: right ventriclular dimension in diastole, TAPSE: tricuspid annular plane systolic excursion, S_R_: lateral wall systolic contractility, PAAT: pulmonary artery acceleration time, PAET: pulmonary artery ejection time (SHAM n = 19; MI with WMSI ≥ 2 n = 18). E/e’: ratio of mitral peak velocity of early filling to early diastolic mitral annular velocity.

### Hemodynamic and morphometric assessment of LV dysfunction and RV hypertrophy and pulmonary hypertension in mice with WMSI ≥2.0

As expected, a lower left ventricular systolic pressure (LVSP) was measured in mice following coronary ligation, as well as an increased left ventricular end diastolic pressure (LVEDP). LV inotropy (+dP/dt) and lusitropy (−dP/dt) were reduced in MI mice with a WMSI ≥2.0. In contrast to their sham counterparts, development of PH was evidenced by an elevated right ventricular systolic pressure (RVSP) concomitantly with RV hypertrophy (RV weight/(LV + septum) weight) (Table 2).

**Table 2:** Morphometric and hemodynamic evaluation of ventricles in SHAM and MI mice.

**Parameters**	**Sham n = 9–10**	**MI (WMSI < 2) n = 3–5**	**p value (WMSI < 2 vs sham)**	**MI (WMSI ≥ 2) n = 12–15**	**p value (WMSI ≥ 2 vs sham)**	**p value (WMSI < 2 vs WMSI ≥ 2)**
***Scar***						
Scar surface (mm²)	NA	11.65 ± 6.4		26.51 ± 1.8		<0.001
Scar weight (mg)	NA	0.0096 ± 0.004		0.015 ± 0.001		<0.05
***Left ventricle***						
LV Weight (g)	0.06 ± 0.003	0.056 ± 0.009	n.s.	0.063 ± 0.005	n.s.	n.s.
(LV + septum) weight (g)	0.079 ± 0.004	0.082 ± 0.006	n.s.	0.09 ± 0.006	n.s.	n.s.
LV/BW	0.0026 ± 0.0002	0.0023 ± 0.0007	n.s.	0.0027 ± 0.0002	n.s.	n.s.
Systolic pressure (mmHg)	117 ± 10	103 ± 11	n.s.	93 ± 4	<0.05	n.s.
LVEDP (mmHg)	10 ± 2	9.8 ± 2	n.s.	18 ± 2	<0.05	n.s.
+dP/dt (mmHg/sec)	8476 ± 767	7568 ± 1149	n.s.	5649 ± 253	<0.01	n.s.
dP/dt (mmHg/sec)	−7591 ± 484	−6725 ± 832	n.s.	−4861 ± 289	<0.001	n.s.
***Right ventricle***						
RV Weight (g)	0.022 ± 0.0008	0.019 ± 0.002	n.s.	0.028 ± 0.003	n.s.	n.s.
RV/(LV + septum) weight (%)	15.27 ± 4.58	23.95 ± 4.9	n.s.	33.46 ± 3.7	<0.05	n.s.
RV/BW	0.00087 ± 0.00008	0.00077 ± 0.00007	n.s.	0.0013 ± 0.0001	<0.001	n.s.
Systolic pressure (mmHg)	24.32 ± 1.2	29.35 ± 0.4	n.s.	31.53 ± 3.2	<0.05	n.s.
RVEDP (mmHg)	5.06 ± 0.8	3.63 ± 0.6	n.s.	2.16 ± 0.5	<0.05	n.s.

LVEDP: left ventricular end diastolic pressure, +dP/dt and −dP/dt: maximum and minimum rates of pressure change (indices of contractility and relaxation), RV: right ventricle, LV: left ventricle, RVEDP: right ventricular end diastolic pressure, BW: body weight.

### Mice respiratory function post-MI

**Figure 4:**
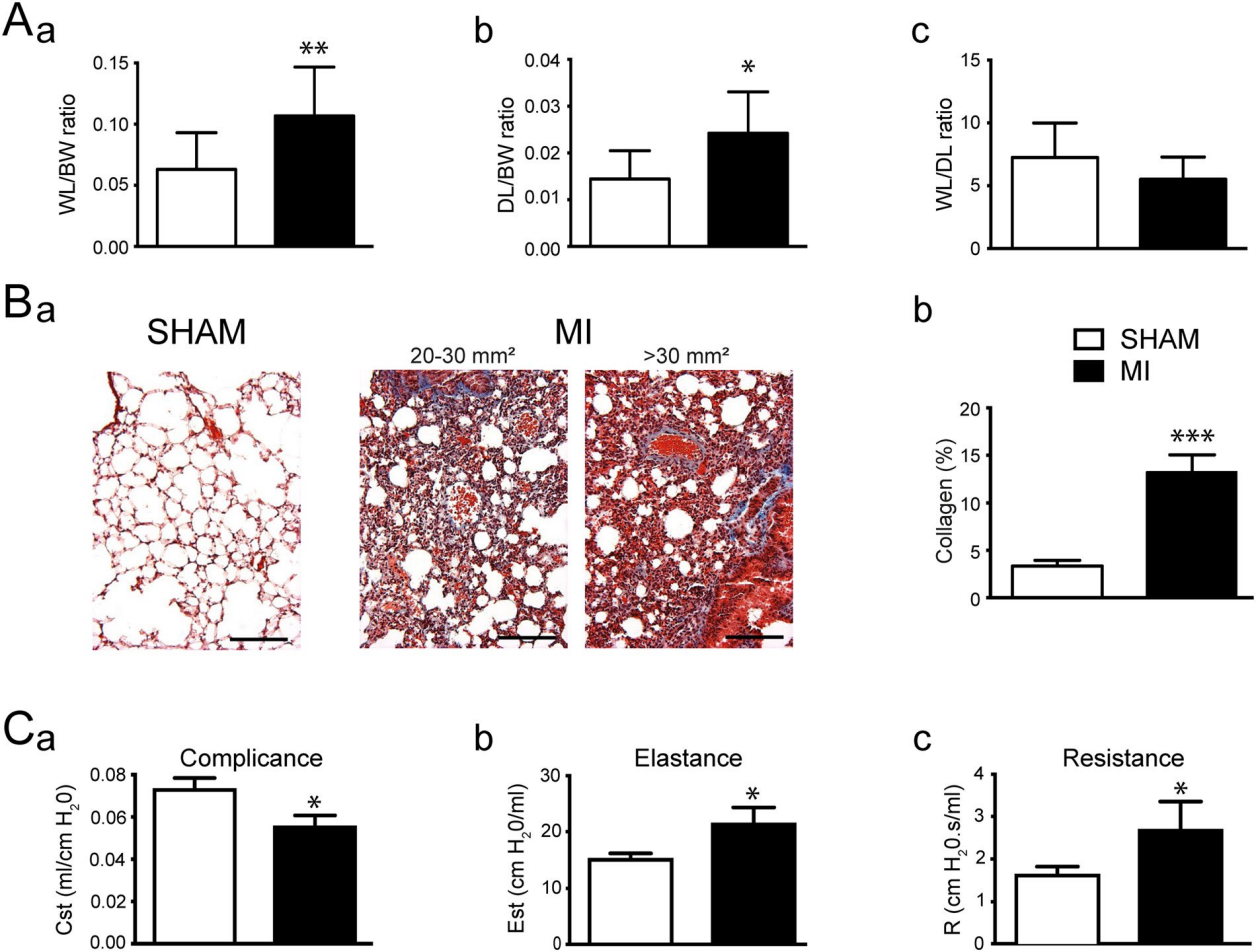
Lung remodelling upon coronary ligation. (**A**) Assessment of pulmonary oedema in mice without (**SHAM**, white) or with myocardial infarct (MI, black). Ratioed (**a**) wet lung and body weights (Sham n = 6; MI n = 15), (**b**) dry lung and body weights (Sham n = 6; MI n = 11), and (**c**) wet and dry lung weights (Sham n = 5; MI n = 14). (**Ba**). Typical examples of pulmonary structural remodelling in mice without (SHAM, white), or with medium (20–30 mm^2^) or large (>30 mm^2^) myocardial infarct (MI, black) revelaed by Masson trichrome stain (20×). (**Bb**). Bar graph reporting collagen deposit in mice without (SHAM, white) and with myocardial infarct (MI, black) measured in experiments as in Ba. n = **C**. Bar graphs reporting respiratory function evaluation in mice without (SHAM, white) or with myocardial infarct (MI, black). (**Ca**). Quasi-static compliance (Cst) (**Cb**). Quasi-static elasticity (Est) (**Cc**). Resistance (R). SHAM n = 7; MI n = 10. *p < 0.05, **p < 0.01 and ***p < 0.001 vs SHAM.

**Figure 5:**
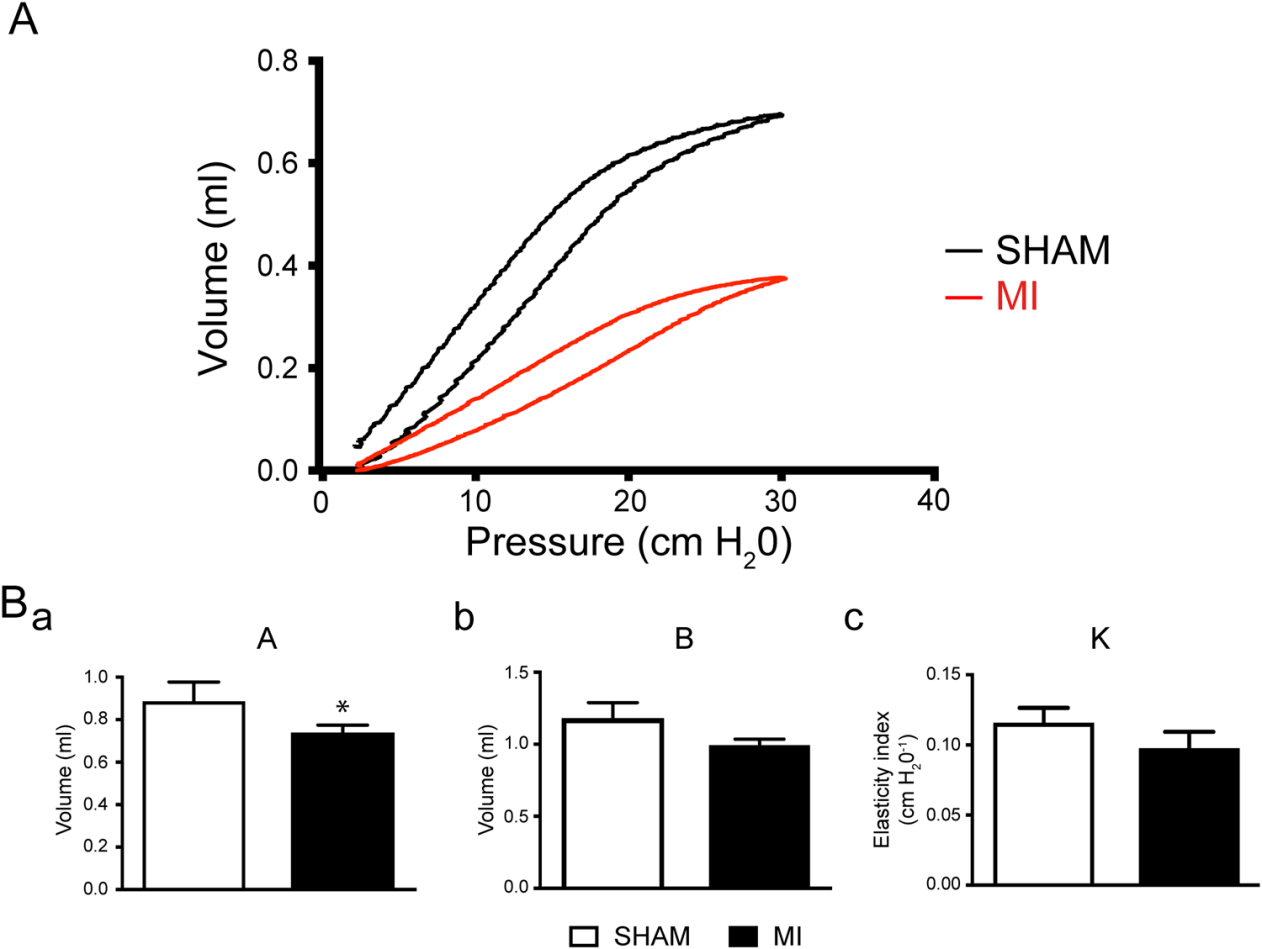
Pulmonary pressure-volume curves after coronary ligation. (**A**) Typical example of pulmonary pressure-volume curves recorded from mice without (SHAM, black) and with myocardial infarct (MI, red). (**B**) Bar graph reporting pulmonary capacity in mice without (SHAM, white) and with myocardial infarct (MI, black) calculated from experiments as in A using the Salazar-Knowles equation. **Ba**. Total inspiratory capacity (**A**) **Bb**. Difference between the total pulmonary capacity and the volume at zero pressure (**B**) **Bc**. Curvature parameter (K). SHAM n = 7; MI n = 10. *p < 0.05 vs SHAM.

Following hemodynamic recordings, respiratory function was assessed to evaluate the impact of MI on pulmonary capacity (Fig. 4). Figure 4A and B illustrate the MI-induced pulmonary alveolar and vascular remodelling evidenced by collagen deposition, as well as cellular proliferation as seen previously in other animal models of myocardial infarction. According to previous work that had been done on a rat model of myocardial infarction, proliferating cells are composed principally of myofibroblasts^9,28^. Interestingly, and similar to the rat MI model, mice with MI and moderate to large infarcts did not display pulmonary oedema (Fig. 4Ac). However, MI mice clearly developed a restrictive respiratory syndrome characterized by a decrease in lung compliance (Fig. 4C) evidenced by a shift in the pressure-volume relationship compared to the sham group (Fig. 5).

### Correlation between WMSI and PH markers in mice with WMSI ≥2.0

**Figure 6:**
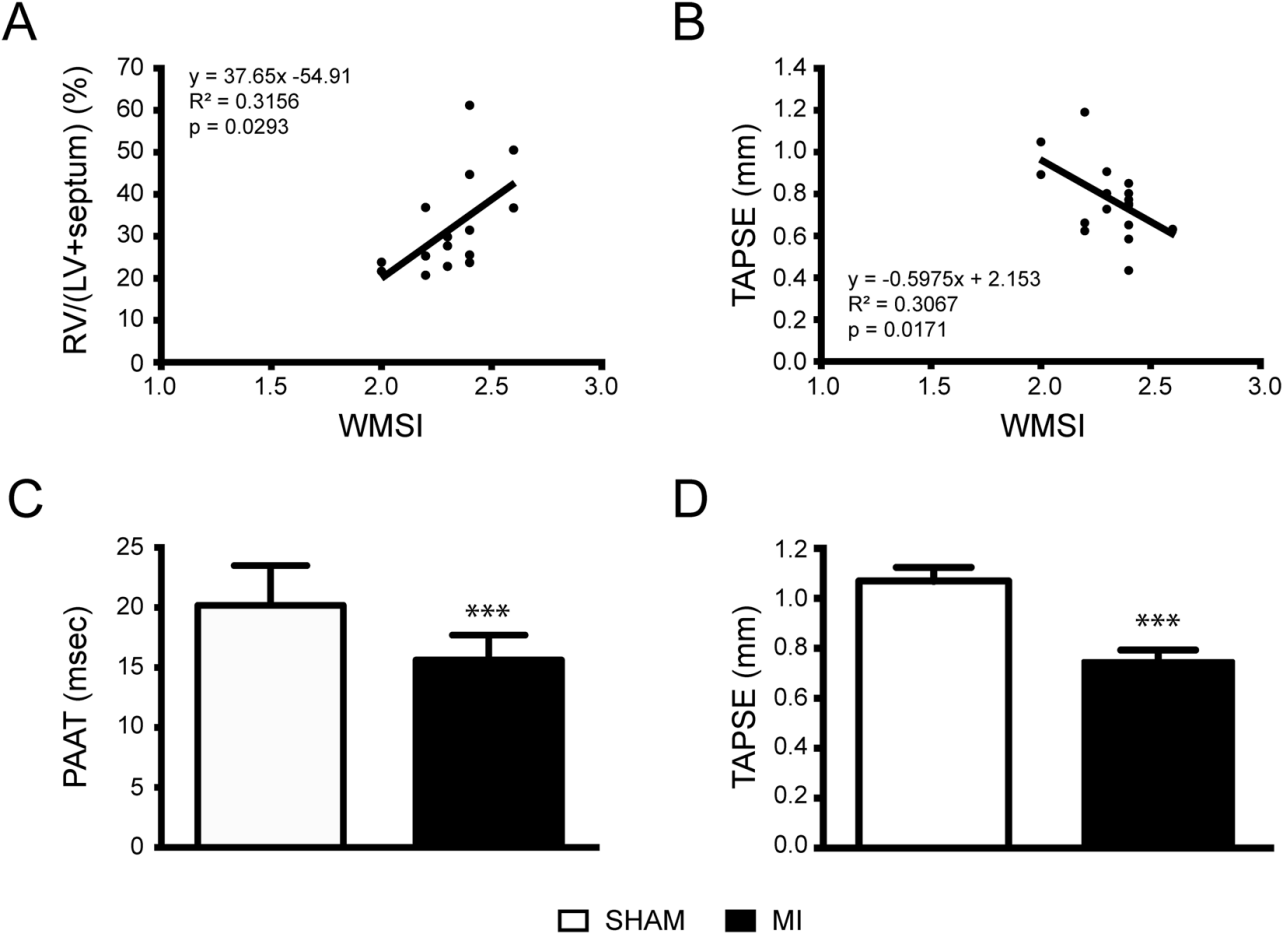
Right ventricle and pulmonary artery following myocardial infarct with a WMSI ≥ 2. (**A**) Correlation of right ventricle hypertrophy with Wall Motion Score Index (WMSI) in mice following myocardial infarct. n = 15 (**B**) Correlation between tricuspid annular place systolic excursion (TAPSE) in mice following myocardial infarct. n = 18 (**C**,**D**). Bar graph illustrating the impact of left ventricular infarct on pulmonary artery acceleration time (PAAT, panel C) and tricuspid annular place systolic excursion (TAPSE, panel D) in mice with a WMSI ≥ 2. SHAM n = 19; MI n = 18. ***p < 0.001 vs Sham.

RV hypertrophy measured by the Fulton index correlated (R^2^ = 0.3156; p = 0.0293) with WMSI (Fig. 6A). Furthermore, TAPSE was inversely correlated with WMSI (R^2^ = 0.3067; p = 0.0171) (Fig. 6B). In addition, pulmonary arterial blood velocity and TAPSE values were significantly decreased in mice with large or medium infarcts when compared to sham animals (Fig. 6C and D). Moreover, ROC curves (Supplemental Fig. 2) were constructed to determine the precision of WMSI to predict left ventricular dysfunction (LVEDP > 10 mmHg) and right ventricular dysfunction (TAPSE < 1.0 mm) using mean values from the sham groups as cut-offs. The precision to predict both RV and LV dysfunction was good with areas under the ROC curves greater than 0.7 for both LVEDP and TAPSE. Accordingly, a WMSI of ≥2.0 predicts LV dysfunction with a sensitivity of 57%, a specificity of 86% and a likelihood ratio of 4.0. RV dysfunction is predicted with a sensitivity of 53%, a sensitivity of 83% and a likelihood ratio of 3.2.

## Discussion

In this study, we demonstrated that mice with large surgically-induced MI develop pulmonary hypertension with lung remodeling and right ventricular dysfunction. We validated the use of echocardiography as non-invasive method of evaluating the severity of both LV and RV function in this small rodent model. More importantly, we validated the use of the WMSI as a reliable marker of infarct size that can be used to predict the development of PH and RV dysfunction.

There is a growing clinical necessity for specific therapies targeting PH associated with LHD (group II PH). The core of pre-clinical investigations resides in the use of a validated animal model for group II-PH that replicates human pathophysiology progression and phenotype. With the development of PH-selective therapies, evaluation of these drugs in properly phenotyped pre-clinical models of group II PH is warranted^29,30^. Myocardial infarct following coronary ligation is unequivocally the most clinically relevant model for group II-PH^11,14,15,31^, but assessment of actual PH and thus right ventricular dysfunction development essentially relies on hemodynamic measures using intraventricular catheters. However, in small rodents like mice, this approach is technically challenging due to the narrow right ventricle, even with expert hands and the smallest high-fidelity catheter available. Moreover, manipulations of weakened animals to introduce the catheter often result in blood loss and although less frequent, lethal hemorrhage. In the absence of RV function monitoring and the capacity to determine PH severity, planification of studies using these tissues becomes difficult. Furthermore, evaluation of novel therapies is impossible in the absence of adequate methods of assessing animals prior to therapeutic allocation. MI-induced PH is usually studied 4–5 weeks following surgery to allow development of PH, without tangible indications of the severity of MI and/or forthcoming PH until terminal hemodynamic assessment of cardiac function. Monitoring of disease progression to study potential treatments or therapeutic approaches is therefore needed.

These technical limitations and a substantial early post-surgical mortality rate are the main reasons the rare use of this model in mice despite growing needs for a better understanding of the mechanisms involved in the progression of the disease. The use of transgenic mice models for group II-PH investigation is desirable to accelerate knowledge acquisition. This study presents and validates in mice an approach commonly used in humans and larger animals to assess cardiac function and establish pulmonary hypertension. The lung histopathological changes found in mice are similar to those previously described in other models of chronic heart failure and in humans^28,32^ and are characterized by an increase in dry lung weight with no significant pulmonary edema. Detailed histologic evaluations in rats and in man revealed that alveolar septa myofibroblasts proliferation is greatly responsible for the increased cellularity with collagen and interstitial matrix deposition^10,11,33^. Echocardiography has been previously used in murine models of cardiomyopathy but not to validate PH in a mouse model of heart failure with reduced ejection fraction^34–38^. Echocardiography is advantageous over cardiac catheterization, even in small animals like mice. This non-invasive technique is reproducible and can be repeated to monitor disease progression. Furthermore, we show that the WMSI is a reliable marker for infarct size and PH in mice. Correlation analysis of WMSI and LV dysfunction indicated a threshold WMSI of 2 for medium and large infarcts. Mice with WMSI ≥2.0 developed PH, RV dysfunction and lung remodelling with a restrictive respiratory physiology. Moreover, WMSI is shown to have good precision to predict both LV and RV dysfunction as assessed by LVEDP and TAPSE. Interestingly, WMSI correlates with infarct size and does not vary over time as shown previously^39^. Therefore, assessment of WMSI can be performed as early as 48 hr following coronary ligation and conservative prediction of the forthcoming PH severity can be made. This is a very important asset for early echocardiographic determination of PH. Since infarct size can be quite variable depending on surgical expertise, it allows to promptly discard animals with small or no infarcts that would not develop LV dysfunction and PH. More importantly, it would allow adequate randomization of animals to test and monitor impact of novel therapeutic approaches on PH evolution.

Animal groups were defined based on MI scar surface as an indicator of infarct severity. A close correlation was found between scar size and WMSI, where a WMSI of 2.0 and higher was associated with medium (20–30 mm^2^) and large (>30 mm^2^) infarcts. LV dysfunction was therefore associated with a threshold WMSI value of 2.0, revealing a minimal scar surface of 20 mm^2^. Additionally, WMSI correlates with altered EF and LVEDP as well as LV inotropy and lusitropy. Although LV dysfunction is usually determined with classical intraventricular catheters, WMSI can be considered as a stand-alone reliable marker for LV dysfunction.

PH was determined by alteration of pulmonary artery blood flow, RV dysfunction and hypertrophy. Fulton’s index and TAPSE values, respectively indicators of RV hypertrophy and function correlate with WMSI herein establishing the latter as a predictive marker of hypertrophied and dysfunctional RV and the consequent PH. RV function decline was also noted through PAAT and TAPSE in mice with medium to large infarcts. Since the WMSI is a measure of infarct size, its correlation with numerous severity parameters of both LV and RV function, many of them known to be co-linear isn’t surprising. Other factors besides WMSI, such as the presence and severity of mitral regurgitation, will impact on the lung circulation and the development of PH. Nevertheless, a WMSI value of 2.0 and above appears as a reliable indicator and predictive marker for RV dysfunction, hypertrophy as well as the ensuing development of PH. The contrary however needs to be interpreted with caution and a WMSI <2.0 was not associated with the absence of PH. Indeed, and despite the small sample size, some animals with WMSI <2.0 had abnormal echocardiographic parameters suggestive of significant PH such as lower PAAT and higher RVAWd, and higher RVSP at cardiac catheterization.

The WSMI is a recognized measure of LV function. As expected, we found a strong inverse correlation between the WMSI and LVEF (r = −0.822, p < 0.0001). Previous human studies have validated the use of the WMSI after MI to predict adverse outcomes. In 144 patients after MI, a predischarge resting WMSI ≥1.50 was superior to LVEF ≤40% to identify patients at risk of cardiac death, unstable angina, nonfatal reinfarction, and HF^40^. In 767 MI patients, WMSI was also an independent predictor of death and HF hospitalization, whereas LVEF was not^41^. Reasons for this discrepancy is unclear but could be explained by compensatory hyperkinesia of normal myocardial segments in the acute phase leading to LVEF overestimation. Furthermore, a comparative study of WMSI determined by cardiac ultrasound and LVEF measured by the Simson’s rule, to LVEF measured by magnetic resonance imaging in 111 patients found that Simpson’s rule overestimated LVEF compared to CMR leading the authors to conclude that the WMSI was a simple, accurate and reliable alternative to the measurement of left ventricular function^42^. We choose the WMSI as a parameter of infarct size for its ease of measurement, its absence of variability in controls (value of 1) and its stability over time. Athought we did not petform a comparative analysis, the very good correlation of WMSI with LVEF however suggests that LVEF could also be used to predict future development of PH and RVH.

In summary investigations and pathophysiological understanding of group II-PH have been hindered by the lack of a validated pre-clinical murine model and protocol. We show that MI from coronary artery ligation is a robust and now validated mouse model of WHO group II PH, reproducing the most frequent aetiology of human PH, i.e. HFrEF consecutive to myocardial infarction^28^. Moreover, an indicator of infarct size through echocardiography, the WMSI is an accurate predictive marker of PH and ventricular dysfunction. Echocardiography in mice thus appears as a preferential method for the diagnosis and monitoring of group II PH over standard intraventricular catheterization.

## Electronic supplementary material

Supplemental material
