# Supplementary material for: Echocardiographic validation of pulmonary hypertension due to heart failure with reduced ejection fraction in mice
Source: Sci Rep. 2018 Jan 22;8:1363. doi: 10.1038/s41598-018-19625-2 (PMC5778040; doi:10.1038/s41598-018-19625-2)
Supplement: Supplementary file 1 — Supplemental material [file 41598_2018_19625_MOESM1_ESM.pdf]

# Supplementary material

**Echocardiographic validation of pulmonary hypertension due to heart failure with reduced ejection fraction in mice**

Dayeh R. Nour[1,2], Tardif Jean-Claude[1,2], Shi Yanfen[1], Tanguay Mégane[2], §Ledoux Jonathan[1,2,3], §*Dupuis Jocelyn[1,2].

**Affiliations:**
[1] Research Center, Montreal Heart Institute, Quebec, Canada
[2] Departments of Medicine, [3] Pharmacology and Physiology, Université de Montréal, Quebec, Canada

§ Drs Ledoux and Dupuis share senior authorship.

*Corresponding author
Research Center
Montreal Heart Institute
5000 Belanger est, Montreal,
Quebec, Canada, H1T 1C8
Email: dupuisj@me.com
Phone: 514-376-3330 ext. 3542
Fax: 514-376-1355

| | *Sham* | *WMSI<2* | *WMSI≥2* |
|---|---|---|---|
| | *N=19* | *n=7* | *n=17* |
| *Normal wall motion* | *190/190* | *47/70* | *43.5/170* |
| *Hypokinesis* | *0/190* | *9.5/70* | *45.5/170* |
| *Akinesis* | *0/190* | *12.5/70* | *63.5/170* |
| *Dyskinesis* | *0/190* | *1/70* | *17.5/170* |
| *Aneurysms* | *0/190* | *0/70* | *0/170* |

Suppl table 1. Grading of wall motion abnormalities in the study population. 10 segments were analyzed for each animal.

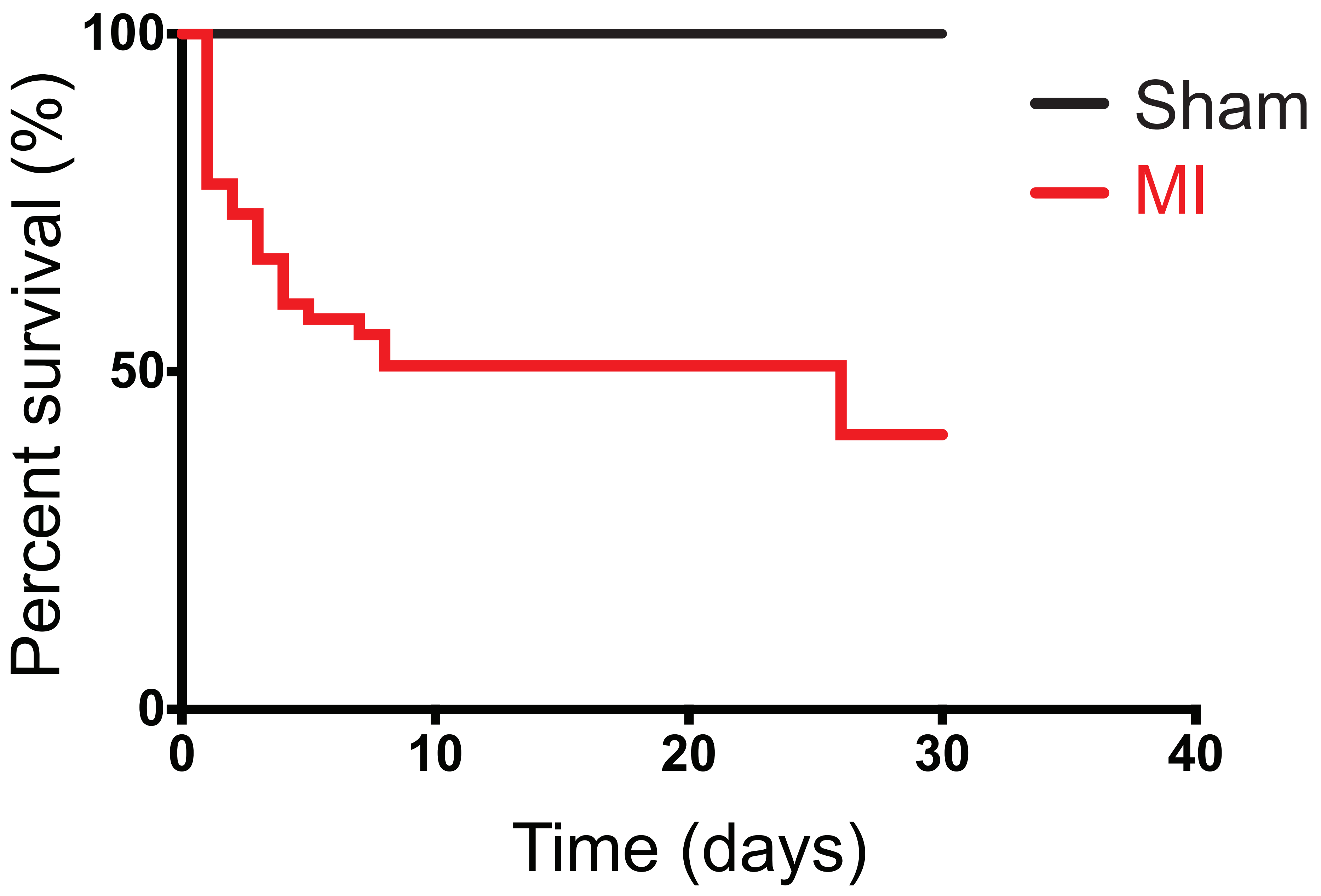

Percent survival (%)
100
50
0
0
10
20
30
40
Time (days)
Sham
MI

A

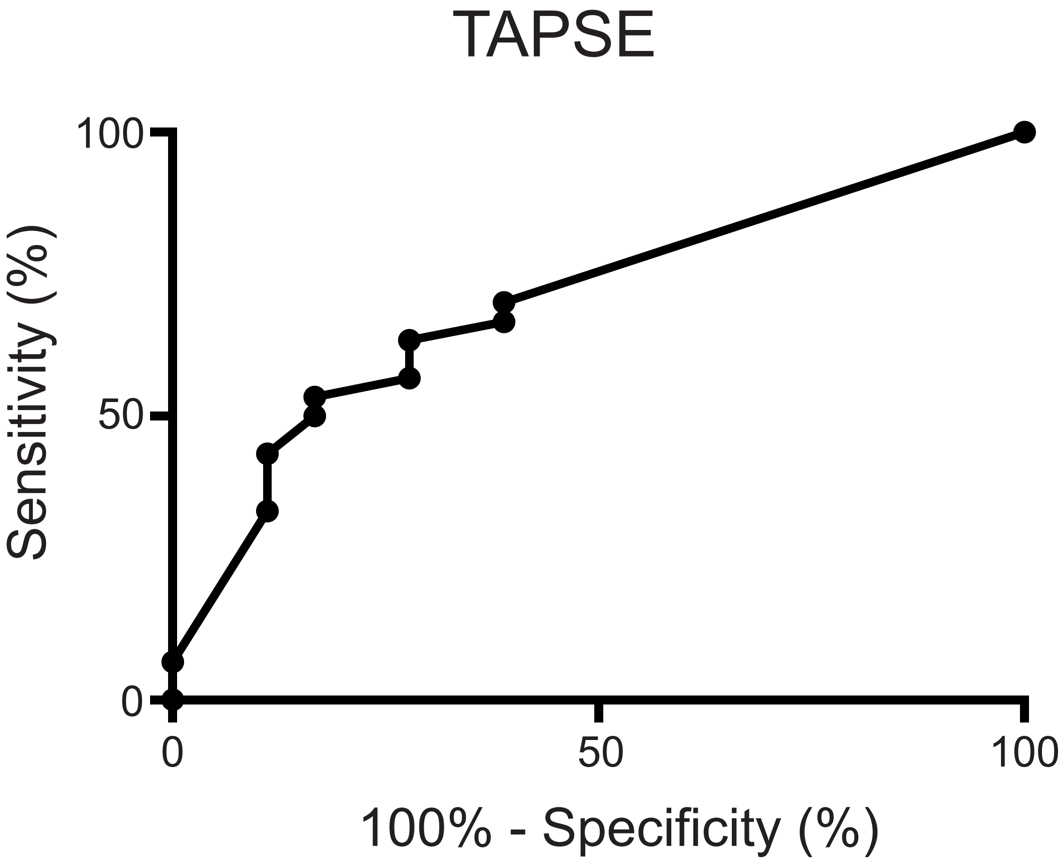
TAPSE
100
50
0
Sensitivity (%)
0
50
100
100% - Specificity (%)

B

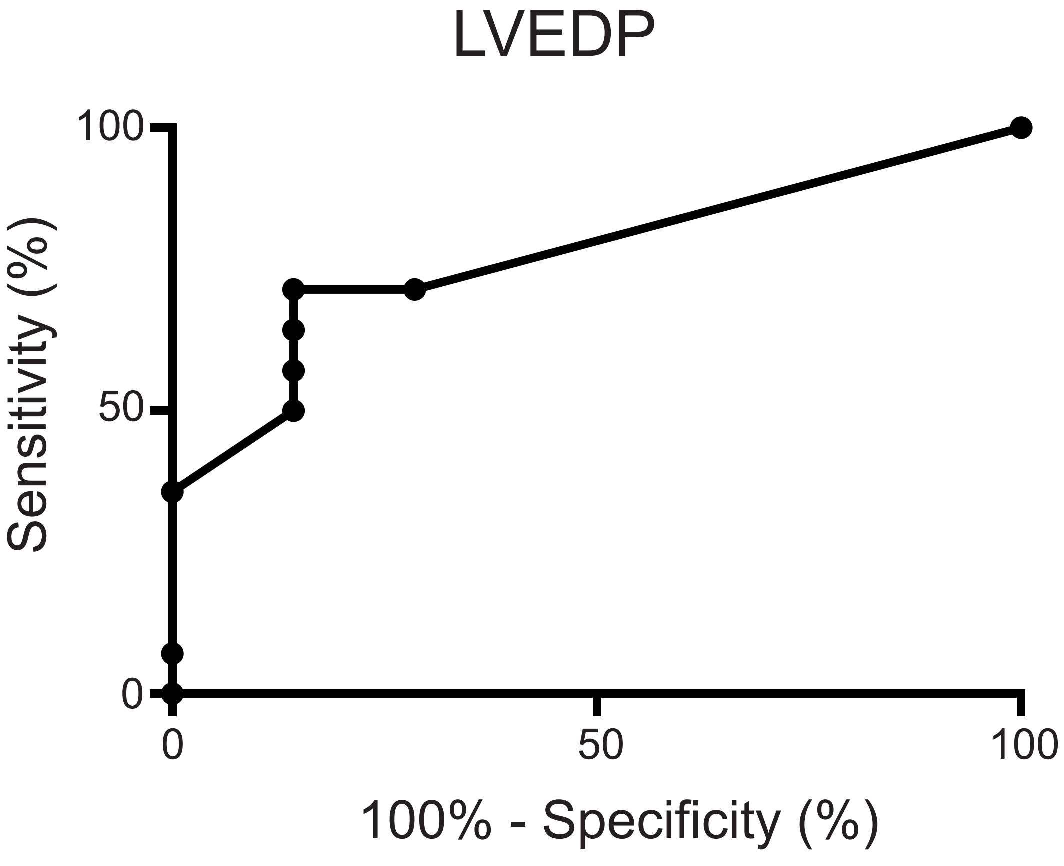
LVEDP
100
50
0
Sensitivity (%)
0
50
100
100% - Specificity (%)

## Supplemental Figures Legends

**Supplemental Figure 1:** *Post MI survival curve*
Kaplan-Meir survival curve for mice following surgical procedure. Mice underwent thoracotomy without (SHAM, black, n= 19)) or with ligation of left anterior coronary artery (MI, red, n=48).

**Supplemental Figure 2:** *ROC curves with WMSI to predict type 2 pulmonary hypertension.*
ROC curves for TAPSE (**A**) and LVEDP (**B**) illustrating the efficiency of WMSI as an early indicator of type 2 pulmonary hypertension in mice. Area under the curve: TAPSE: 0.7009; LVEDP: 0.7755. TAPSE: n = 18 (CTRL) & 30 (PH); LVEDP: n = 7 (CTRL) & 14 (PH).
